# Estrogen Signaling Dictates Musculoskeletal Stem Cell Behavior: Sex Differences in Tissue Repair

**DOI:** 10.1089/ten.teb.2021.0094

**Published:** 2022-08-08

**Authors:** Kelsey E. Knewtson, Nathan R. Ohl, Jennifer L. Robinson

**Affiliations:** ^1^ Department of Chemical and Petroleum Engineering, University of Kansas, Lawrence, Kansas, USA.; ^2^ Bioengineering Graduate Program, University of Kansas, Lawrence, Kansas, USA.

**Keywords:** estrogen, sexual dimorphism, musculoskeletal stem cells, bone marrow mesenchymal stromal cells, adipose derived stem cells

## Abstract

Sexual dimorphisms in humans and other species exist in visually evident features such as body size and less apparent characteristics, including disease prevalence. Current research is adding to a growing understanding of sex differences in stem cell function and response to external stimuli, including sex hormones such as estrogens. These differences are proving significant and directly impact both the understanding of stem cell processes in tissue repair and the clinical implementation of stem cell therapies. Adult stem cells of the musculoskeletal system, including those used for development and repair of muscle, bone, cartilage, fibrocartilage, ligaments, and tendons, are no exception. Both *in vitro* and *in vivo* studies have found differences in stem cell number, proliferative and differentiation capabilities, and response to estrogen treatment between males and females of many species. Maintaining the stemness and reducing senescence of adult stem cells is an important topic with implications in regenerative therapy and aging. As such, this review discusses the effect of estrogens on musculoskeletal system stem cell response in *multiple species* and highlights the research gaps that still need to be addressed. The following evidence from investigations of sex-related phenotypes in adult progenitor and stem cells are pieces to the big puzzle of sex-related effects on aging and disease and critical information for both fundamental tissue repair and regeneration studies and safe and effective clinical use of stem cells.

**Impact Statement:**

This review summarizes current knowledge of sex differences in and the effects of estrogen treatment on musculoskeletal stem cells in the context of tissue engineering. Specifically, it highlights the impact of sex on musculoskeletal stem cell function and ability to regenerate tissue. Furthermore, it discusses the varying effects of estrogen on stem cell properties, including proliferation and differentiation, important to tissue engineering. This review aims to highlight the potential impact of estrogens and the importance of performing sex comparative studies in the field of tissue engineering.

## Introduction

Studies of sex-based differences in humans have traditionally focused on visually evident features, including body size, anatomical differences, and life span. Before the encouragement of the National Institutes of Health (NIH) to include sex as a variable, most studies across mammalian species used solely male specimens. Reasons for this include concerns about complications due to the estrous cycle in females, the pressures of convention, and a lack of understanding of the potential effect of sex on results.^1^ These one-sided studies obscure important sex differences that could otherwise aid in future study design and discoveries. Furthermore, not including both sexes contributes to the lack of reproducibility in preclinical research,^2^ supported by the fact that women experience more adverse drug reactions than men.^3^

**FIG. 1.**
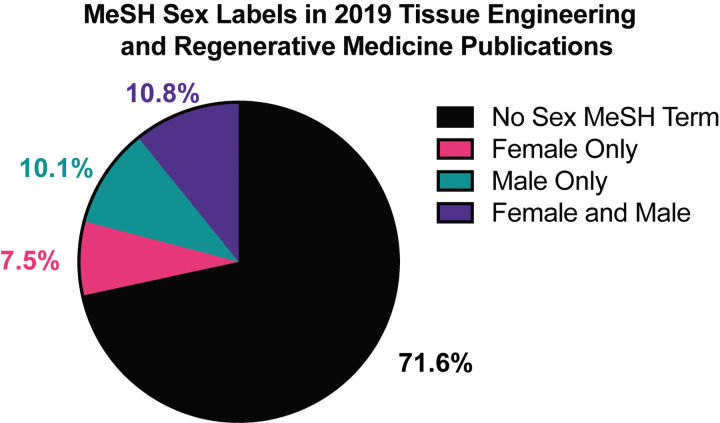
Percentage of tissue engineering and regenerative medicine publications with sex MeSH terms in 2019. A PubMed search was performed using: ““tissue engineering” OR “regenerative medicine” NOT review. PubMed's sex filters were used to determine the numbers of publications tagged with the MeSH terms “Male” and/or “Female” with results limited to 2019 using PubMed's year filter. MeSH, medical subject headings. Color images are available online.

In a PubMed search of tissue engineering and regenerative medicine publications from 2019, only 28.4% of the 10,651 publications reported subject sex at all (Fig. 1). Of that subset of studies, only 38% reported using both male and female samples. Such issues highlight the need for including sex as a variable in preclinical studies, specifically those focused on regenerative therapies.

Sexual dimorphism is seen in many diseases, including those of the musculoskeletal system. The reasons for such differences are manifold, complex, and not completely understood but include differences in joint and muscle anatomy, tissue mechanics, and both levels and signaling mechanisms of sex hormones.^4^ Sex-based differences are seen across a wide range of ages. Henschke *et al.* found a stepwise increase in the rate of musculoskeletal disorders in males and females from age 0 to 14 years, with differences in rates between sexes only appearing in the oldest groups.^5^ A study comparing adolescent athletes aged 12–17 to general population controls found that females in both groups had higher prevalence of symptoms in most body regions compared to age-matched males, while males in both groups had higher prevalence for elbow symptoms.^6,7^

Males and females experience aging in different ways, with sex hormones gradually decreasing as males age but rapidly declining in females during menopause.^8^ Epidemiological evidence illustrates the effect these natural changes in hormone reduction have on tissue homeostasis and function. Wolf *et al.* reviewed musculoskeletal disease rates in males and females^4^ and found differences in rates for many conditions, including joint injuries and osteoporosis. Females, especially after menopause, are more likely to develop osteoarthritis than males, and their disease is typically more severe.^9,10^ Although the reasons for these discrepancies are not fully understood, sex-based differences in cells' response to the microenvironment likely contribute.

Sex-based differences are found in stem cells from various tissues, including those of the musculoskeletal system, and have been shown to affect their therapeutic potential. The inherent ability to self-renew, produce trophic factors to stimulate and organize surrounding cells for repair, and differentiate into mature cell phenotypes makes stem cells a vital component of tissue engineering and regenerative therapies. Differences between sexes have been seen in musculoskeletal stem cell number, proliferation, and differentiation. Differences in patient relapse rates and nonrelapse mortality after allogenic hematopoietic stem cell transplants provide clinical evidence of the importance of stem cell donor sex to regenerative therapy.^11^

Animal studies have also found sex-based differences in the therapeutic potential of stem cells. For example, bone marrow-derived mesenchymal stem cells from female mice better aid in rat cardiac recovery after ischemia and endotoxemia than male cells,^12,13^ but male muscle-derived stem cells (MDSCs) have been found to heal defects in bone and cartilage more effectively.^14–16^ Many, although not all, of these differences have been linked to estrogens. For this reason, this review is focused on estrogen signaling and this hormone class' control of these stem cell processes.

**FIG. 2.**
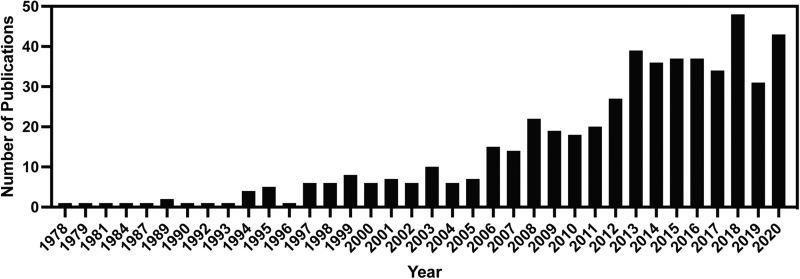
Publications on estrogen and musculoskeletal stem cells by year in PubMed. Search was performed on March 5, 2021 using the following search terms: estrogen AND stem cell AND (bone OR muscle OR adipose OR cartilage OR tendon OR Ligament) AND (proliferation OR apoptosis OR senescence OR viability OR differentiation) NOT (cardiovascular OR cancer OR urogenital system OR hematopoiesis). As of October 2021, there were already 44 publications that fit this search for 2021.

Maintaining the stemness and reducing senescence of stem cells is an important topic with implications in regenerative therapy and aging. The significance of the role sexual dimorphism plays in these processes is highlighted by the prevalence of this topic in previous reviews^17–21^ and the increasing number of publications on the topic (Fig. 2). The current review provides an updated and focused compilation of the effects of estrogens, most often 17β-estradiol (E2), on musculoskeletal stem cell processes critical for tissue engineering and stem cell therapies. The goal of this review is to compile what is known and highlight the research gaps that still need to be addressed to advance tissue engineering and regenerative therapies.

## Sex-Based Differences in Musculoskeletal Stem Cells

Sex-based differences in cell number, proliferative ability, and differentiation potential occur in bone marrow-derived mesenchymal stromal cells (BM-MSCs), adipose-derived stem cells (ASCs), and MDSCs from *multiple species*. In general, males have more BM-MSCs with higher differentiation potential than females, although sex is typically not a factor in proliferation. Fewer BM-MSCs have been found in the bone marrow of female mice^22^ and rats^23^ than in males. It is hypothesized that this difference in progenitor cell number contributes to the ability of male rats to heal more efficiently after femoral bone defects than female rats.^23^ Katsara *et al.* found that male mouse BM-MSCs showed stronger osteogenic and adipogenic potential than female cells, although sex did not affect proliferative abilities.^22^ Similarly, no sex-related differences were seen in proliferation or senescence in BM-MSCs isolated from rats.^23^

Li *et al.*, although, found that female smooth muscle progenitor cells derived from both embryonic stem cell lines and induced pluripotent stem cells were more proliferative than their male counterparts. Furthermore, female progenitor cells derived from induced pluripotent stem cells expressed more estrogen receptor β (ERβ) than male cells, but both sexes expressed equivalent levels of ERα.^24^ Depending on the tissue source and study design, nuclear ER expression levels have been shown to either be comparable or differentially expressed comparing ERα and ERβ between male and female cells. As such, it is not clear whether ER expression levels are a major factor in sex-related differences observed in stem cell behavior.

Sexual dimorphism in differentiation is seen in human ASCs. Aksu *et al.* found that male ASCs showed greater osteogenic differentiation compared to cells from female patients.^25^ Bianconi *et al.* used Transcriptome Mapper to analyze gene expression data for human ASCs from the Gene Expression Omnibus. Analysis of data from 12 males and 33 females between 18 and 71 years old revealed many chromosomal segments, and individual genes were differentially expressed between the sexes, including some related to differentiation.^26^

In general, male MDSCs have greater differentiation capabilities than female cells. *In vitro* studies show that MDSCs isolated from male mice have greater osteogenic^27^ and chondrogenic^14^ potential than those isolated from female mice. Similar differences were seen in human MDSCs, with male cells having greater chondrogenic and osteogenic potential than female cells.^16^ Deasy *et al.* found that male mouse MDSCs differentiate more after oxidative stress, potentially leading to a quicker depletion of the stem cell population than is seen for female cells.^28^

As seen in *in vitro* studies, *in vivo* studies reveal sex differences in differentiation potential, with male cells having the greater ability to regenerate tissue in most reports. Several studies have explored the regenerative capabilities of mouse MDSCs genetically engineered to express bone morphogenetic protein 4 (MDSC-BMP4). Male MDSC-BMP4 cells were better able to generate ectopic bone in sex-matched mice^27^ and articular cartilage in female rats^14^ than female MDSC-BMP4 cells. In a similar study, male mouse MDSC-BMP4 cells were implanted ectopically and into cranial defects in both unaltered and gonadectomized male and female mice. For both types of implants, male hosts showed greater bone formation than female hosts.^15^

Similar sex differences are seen in the bone-forming capabilities of human MDSCs genetically engineered to express bone morphogenetic protein 2 (hMDSC-BMP2) as were seen in mouse MDSCs. When hMDSC-BMP2 cells were implanted into calvarial bone defects in mice, cells of both sexes were able to regenerate bone, but male cells did so more efficiently.^16^ Conversely, Deasy *et al.* found that MDSCs from female mice regenerated skeletal muscle in mice more efficiently than cells from males.^28^ Overall, male musculoskeletal stem cells exhibit enhanced differentiation capacity compared to female cells.

## Estrogens and Musculoskeletal Stem Cells

**FIG. 3.**
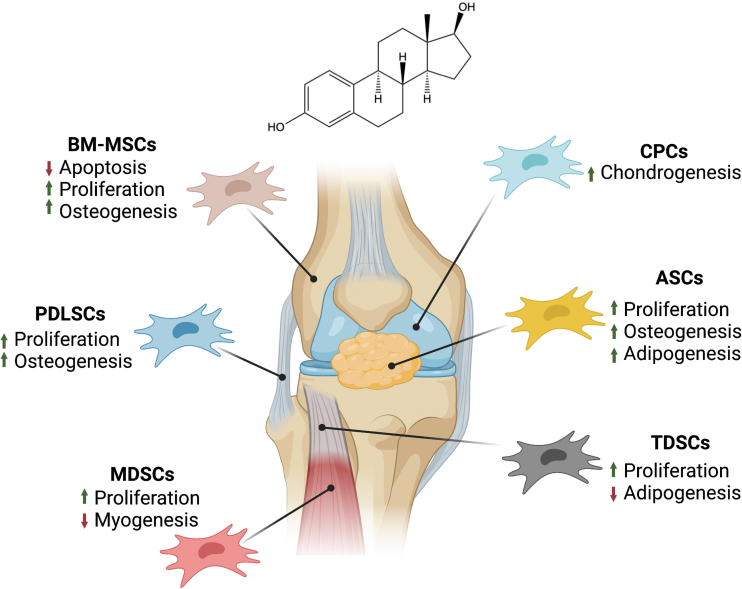
Summary of the effects of E2 on stem cells of the musculoskeletal system. The knee is used as a placeholder for other tissues due to the fact that it contains all tissue types of interest. Effects shown are the general trends for each cell type given the information presented in this review. Figure created using BioRender.com ASCs, adipose-derived stem cells; BM-MSCs, bone marrow-derived stromal cells; CPCs, chondrogenic progenitor cells; E2, 17β-estradiol; MDSCs, muscle-derived stem cells; PDLSCs, periodontal ligament stem cells; TDSCs, tendon-derived stem cells. Color images are available online.

In the following sections, studies are divided first according to stem cell phenotype. Where applicable, they are then subdivided into *in vitro* and *in vivo* studies and again by species. Some studies are discussed in multiple sections. General trends are illustrated in Figure 3. Tables 1–4 contain summaries of all reviewed studies.

**Table 1.** Role of Estrogens on Bone Marrow-Derived Stromal Cell Stemness

Cell type	Bone marrow derived stromal cells					
	Sex	Animal model, age	In vitro, in vivo	Hormone treatment	Response	Study
Mouse cells						
BM-MSCs	F and M	C57 mice, 8-week old	*In vitro*	None	After stress by LPS treatment or hypoxia: F: increased VEGF compared to M M: increased TNF and IL-6 compared to F; hypoxia induced more apoptosis compared to F	Crisostomo *et al.*^12^
BM-MSCs	F and M	BALB/c mice of different ages	*In vitro*	None	F and M: higher passages decreased adipogenic potential and increased osteogenic potential M: greater potential toward both adipogenic and osteogenic lineages compared to F	Katsara *et al.*^22^
BM-MSCs	F	C57/BL6 mice, 8-week old	*In vitro*	ovx	ovx: increased adipogenic markers; decreased osteogenic markers	Qi *et al.*^31^
BM-MSCs	F	Swiss-Webster mice, 7-month old	*In vitro*	10^−10^–10^−6^ M E2; ovx	E2 treatment: increased proliferation and differentiation to osteoblasts; decreased apoptosis; increased the expression of ERα; decreased the expression of ERβ ovx: proliferation and differentiation were lower than in cells from sham mice; apoptosis was higher.	Zhou *et al.*^29^
Bone marrow-derived stroma cell line ST2 stably overexpressing human ERα or ERβ	N/A	Mouse, age N/A	*In vitro*	0–1 nM E2	Cotreatment of cells with estrogen and (BMP)-2: increased osteogenesis compared to cells treated with just BMP-2 Treatment with E2: decreased adipogenesis	Okazaki *et al.*^33^
BM-MSCs	N/A	C57BL/6 mice, age N/A	*In vitro*	10 nM E2	E2 treatment: increased osteogenesis	Pang *et al.*^32^
BM-MSCs	F	C57BL/6 mice, 8-week old	*In vitro*	10^−7^ M E2; ovx	ovx mice: BM-MSCs more senescent, less proliferative, and lower osteogenic potential than those from sham animals; these deficiencies were alleviated by E2 treatment; effects linked to the JAK2/STAT3 pathway.	Wu *et al.*^30^
Rat cells						
BM-MSCs	F and M	Sprague-Dawley rats, 12-month old	*In vitro*	None	No sex related differences in proliferation, differentiation, or senescence F: fewer BM-MSCs compared to M M: MSCs showed superior healing compared to F	Strube *et al.*^23^
BM-MSCs	F	Sprague-Dawley rats, 12-week old	*In vitro*	ovx	ovx: reduced pluripotency and increased senescence through ERβ-SATB2	Wu *et al.*^34^
BM-MSCs	F and M	F-344 rats, 3-month old	*In vitro*	10^−6^–10^−12^ M E2	E2 treatment: F: lower concentrations increased proliferation rate and osteogenic potential M: no change in proliferation rate; increased osteogenic potential	Hong *et al.*^36^
BM-MSCs	F	Sprague-Dawley rats, 9-week old	*In vitro*	10^−7^ M E2; ovx	E2 treatment: increased colony numbers and number of cells per colony of cells; no effect on osteogenic potential; decreased adipogenic potential; decreased apoptosis	Ayaloglu-Butun *et al.*^35^
BM-MSCs	F	Sprague-Dawley rats, 8-week old	*In vitro*	1 nM E2	E2 treatment: increased number of cells in the S-phase; increased osteogenic differentiation; decreased chondrogenesis activated MAPK pathway	Zhao *et al.*^37^
BM-MSCs	M	Sprague-Dawley rats, 4-week old	*In vitro*	0, 1, 10, 100, 500, 1000 nM E2	E2 treatment: no effect on viability; dose-dependent increases in osteogenesis.	Liu *et al.*^38^
Larger animal cells						
BM-MSCs	F and M	Mini-pig, 1-year old	*In vitro*	0, 10^−6^, 10^−8^, 10^−10^, 10^−10^, 10^−12^, 10^−14^ M E2	E2 treatment: reduced apoptosis-related gene expression and increased chondrogenesis in both sexes F: proliferation rates increased with decreasing concentration; increased osteogenic differentiation; decreased adipogenic differentiation M: lower concentrations increased proliferation while higher concentrations decreased proliferation; increased adipogenic differentiation	Lee *et al.*^39^
BM-MSCs	M	Beagle dogs, sexually mature	*In vitro*	0, 10^−7^, 10^−9^, 10^−11^, 10^−13^, 10^−15^ M E2	E2 treatment: Above 10^−11^ M: inhibited proliferation and increased apoptosis 10^−11^ M: increased proliferation; decreased apoptosis; no effect on osteogenesis or adipogenesis.	Zhou *et al.*^40^
Human cells						
BM-MSCs	F	Human, 41–51-year old, perimenopausal	*In vitro*	10 nM E2	Osteogenic differentiation: ERα and ERβ expression increased Adipogenic differentiation: ERα expression increased; ERβ unchanged E2 treatment: increased osteogenesis; decreased adipogenesis	Heim *et al.*^48^
BM-MSCs	M	Human, 40–44-year old	*In vitro*	1, 2, 4, 8, 10, 50, 100 nM E2	E2 treatment: increased osteoblast proliferation in a dose dependent manner between 1 and 8 nM, with no further increase seen at higher concentrations; 1 and 2 nM E2 increased proliferation, but higher doses had no effect.	DiSilvio *et al.*^41^
BM-MSCs	M	Human, adult	*In vitro*	10 nM, 10 pM E2	E2 treatment + osteogenic stimulation: enhanced osteogenic potential; no change in proliferation E2 treatment + adipogenic stimulation: increased adipogenic potential, decreased proliferation	Hong *et al.*^45^
BM-MSCs	M	Human, 18–45-year old	*In vitro*	10^−11^–10^−8^ M E2	E2 treatment: no effect on proliferation; inhibited chondrogenesis	Jenei-Lanzl *et al.*^44^
BM-MSCs	M	Human, 31–62-year old	*In vitro*	10^−7^, 10^−9^, 10^−11^ M E2	E2 treatment: no effect on cell proliferation rate, time to senescence, or the expression of telomere and senescence-associated genes; decreased telomere shortening over time	Breu *et al.*^43^
BM-MSCs	F and M	Human, 27.4 ± 6.1-year old	*In vitro*	10^−6^–10^−12^ M E2	E2 treatment: increased proliferation in both sexes; maintained proliferation rates through more passages than control cells; increased ERα expression; ERβ expression unchanged.	Hong *et al.*^42^
BM-MSCs	F	Human, age N/A	*In vitro*	100 pM–1 mM E2	E2 treatment: increased osteogenic and adipogenic potential	Strong *et al.*^47^
BM-MSCs	N/A (lot specific)	Human, age N/A (lot specific)	*In vitro*	1 nM E2	E2 treatment: upregulated expression of components of autophagosome genes; increased autophagic flux	Gavali *et al.*^46^

BM-MSC, bone marrow-derived mesenchymal stromal cell; BMP-2, bone morphogenetic protein 2; E2, 17β-estradiol; ERα, estrogen receptor α; ERβ, estrogen receptor β; F, female; IL-6, interleukin 6; LPS, lipopolysaccharide; M, male; MAPK, mitogen-activated protein kinase; N/A, not available; ovx, ovariectomized; TNF, tumor necrosis factor; SATB2, sequence binding protein 2; VEGF, vascular endothelial growth factor.

## Estrogens and BM-MSCs

### Mouse cells

E2 treatment has effects on apoptosis rates, proliferation, and ER expression of BM-MSCs from mice. BM-MSCs isolated from ovariectomized (ovx) mice were more apoptotic,^29^ senescent,^30^ and less proliferative^29,30^ than sham controls, and E2 treatment improved these characteristics.^29,30^ E2 treatment also increased the expression of ERα and decreased the expression of ERβ.^29^ Wu *et al.* found that E2 control of proliferation and senescence was linked to the JAK2/STAT3 pathway.^30^

Estrogens are important for BM-MSC differentiation in mice. BM-MSCs isolated from ovx mice showed decreased osteogenic^29–31^ and increased adipogenic^31^ differentiation compared to sham controls. Multiple studies have found that E2 treatment of mouse BM-MSCs increased their osteogenic potential.^29,30,32^ Similarly, Okazaki *et al.* found that E2 treatment of mouse BM-MSCs that overexpress human ERα or ERβ promoted osteogenic differentiation, although it decreased adipogenic potential. In both cell lines, these effects were blocked by the nonspecific ER antagonist ICI 182,780. These data suggest similar roles for both receptors in these processes.^33^

### Rat cells

E2 treatment also affects many cell cycle-related characteristics of rat BM-MSCs. Wu *et al.* found that BM-MSCs from ovx Sprague-Dawley rats exhibited increased senescence compared to sham controls.^34^ Ayaloglu-Butun *et al.* reported that apoptosis rates were similar between BM-MSCs from intact and ovx female Sprague-Dawley rats and decreased with E2 treatment. They also explored the effects of E2 treatment on BM-MSCs from both groups and found that it caused an increase in the colony numbers and number of cells per colony isolated from both groups.^35^ Hong *et al.* reported that E2 treatment increased proliferation in BM-MSCs from female rats but not from males.^36^ A study by Zhao *et al.* revealed that E2 treatment of BM-MSCs isolated from female Sprague-Dawley rats increased the number of cells in the S-phase of the cell cycle compared to controls.^37^

Hormone status and E2 treatment have more complicated effects on differentiation in rat BM-MSCs than in mouse cells. Wu *et al.* found that BM-MSCs from ovx Sprague-Dawley rats exhibited reduced stemness and osteogenic differentiation compared to sham controls. These phenotypes were rescued by E2 treatment through ERβ and special AT-rich sequence binding protein 2 (SATB2) signaling.^34^ Conversely, Ayaloglu-Butun *et al.* saw no differences in the osteogenic or adipogenic potential of BM-MSCs isolated from intact and ovx female Sprague-Dawley rats. Furthermore, E2 treatment had no effect on osteogenic potential but decreased adipogenic potential in this study.^35^

Differences between the two studies are potentially explained by differing differentiation protocols and assay readouts. Several groups report that E2 treatment increases the osteogenic potential of BM-MSCs isolated from male^36,38^ and female^36,37^ rats. Furthermore, one of these studies revealed that E2 treatment decreased chondrogenic potential in female rat BM-MSCs and activated the MAPK pathway.^37^

### Larger animal cells

Lee *et al.* studied the effect of estrogen treatment on BM-MSCs collected from mature mini-pigs of both sexes. The study revealed that 1 pM E2 was the optimum concentration to increase proliferation of cells of both sexes, while 1 and 0.01 μM E2 decreased proliferation in male cells. In addition, E2 treatment reduced apoptosis-related gene expression in both sexes. It also decreased adipogenic differentiation in females but increased it in males, increased osteogenic differentiation in females but had no effect in males, and increased chondrogenic potential, although not significantly, in both sexes.^39^ Zhou *et al.* found that E2 treatment of BM-MSCs isolated from male beagles at concentrations above 10 pM inhibited proliferation and increased apoptosis. Ten pico molar E2 showed some signs of increasing proliferation and decreasing apoptosis, but had no effect on osteogenesis or adipogenesis.^40^

### Human cells

In most studies, treatment with E2 increased proliferation of human BM-MSCs, although the effect was at times concentration or sex dependent. DiSilvio *et al.* observed that 1 and 2 nM of E2 increased proliferation of male human BM-MSCs, but that higher concentrations had no effect.^41^ Hong *et al.* found that 0.1–10 nM E2 increased the proliferation of BM-MSCs from both sexes, but that 0.001 nM E2 only increased male cell proliferation. In addition, E2 supplementation maintained proliferation rates through more passages than control.^42^

Conversely, other studies have found E2 treatment to have no effect on male BM-MSC proliferation in 2D^43^ (0.01–100 nM) or 3D^44^ (0.01–10 nM) culture. Hong *et al.* found that stimulation of male BM-MSCs with 10 pM or 10 nM E2 had no effect on cell proliferation during osteogenesis, but inhibited proliferation during adipogenesis.^45^ Due to the fact that concentrations of E2 used in these studies generally overlap, other explanations such as differences in cell source and experimental conditions must be considered.

Effects are seen in other aspects of human BM-MSC cycle regulation after treatment with E2 as well. Gavali *et al.* found that E2 treatment of a human BM-MSC line (ATCC PCS-500-012) upregulated expression of two subunits of RAB3 GTPase Activating Protein Complex, which modulates autophagy.^46^ Breu *et al.* studied the effects of E2 treatment on male BM-MSCs and found that it had no effect on time to senescence or the expression of telomere and senescence-associated genes. E2 treatment did decrease telomere shortening over time in a dose-dependent manner, though.^43^

E2 treatment also increases the differentiation potential of human BM-MSCs, although not universally. It was reported to increase both the adipogenic and osteogenic potential of human BM-MSCs isolated from females^47^ and males.^45^ Heim *et al.* reported that E2 increased osteogenesis but decreased adipogenesis in BM-MSCs isolated from perimenopausal females.^48^ Jenei-Lanzl *et al.* found that E2 treatment inhibited chondrogenesis in male human BM-MSCs in 3D culture. This effect was linked to membrane-associated ERs rather than classical intracellular ERs.^44^

Multiple studies have explored the expression of ERs in BM-MSCs. Heim *et al.* found that ERα expression increased during both adipogenic and osteogenic differentiation, but that ERβ expression only increased during osteogenic differentiation in BM-MSCs from perimenopausal females.^48^ Furthermore, Hong *et al.* found that ERα expression in BM-MSCs was increased by E2 supplementation while ERβ expression was unchanged.^42^

E2's effects on BM-MSCs have been investigated by many and cover several species, as reviewed in Table 1. Generally, E2 treatments increased proliferation and decreased apoptosis and senescence of cells from both females and males of the species considered. In most cases, osteogenesis increased and adipogenesis decreased when treated with E2.

## Estrogens and ASCs

### In vitro

#### Mouse cells

Zhang *et al.* used the ERα agonist propyl pyrazole triol (PPT), the ERβ agonist diarylpropionitrile (DPN), and the ERα/β nonspecific antagonist ICI 182,780 to study the roles each ER plays in male mouse ASCs. The study revealed that both ER agonists increased cell proliferation in a dose dependent manner that was blocked by coincubation with the antagonist, but that the effect of the ERα agonist PPT was greater. In addition, only PPT caused a statistically significant improvement in wound healing and migration. When the ASCs were grown in brown adipogenic differentiation media, PPT stimulated brown adipogenesis, while DPN inhibited it.^49^

#### Rat cells

In general, E2 treatment increases rat ASC proliferation. Feng *et al.* found that E2 increased proliferation and myogenic differentiation of Sprague-Dawley ASCs from both sexes grown both in 2D and on an electrospun poly(lactide)/poly(caprolactone) nanofibrous scaffold, suggesting E2 as a promising tool in tissue engineering.^50^ Interestingly, a wider range of E2 concentrations was found to increase cell proliferation in female cells (0.01 nM–0.1 μM E2) than in male cells (0.1 nM–0.1 μM E2),^50^ an effect opposite to that seen in human BM-MSCs.^42^

Studies of preadipocytes from femoral, epididymal, and parametrial tissue of intact male and intact and ovx female Sprague Dawley rats by Dieudonne *et al.* revealed that 10 nM E2 treatment had no effect on the proliferation of male cells from any studied tissue but did increase proliferation in femoral cells from both ovx and intact females.^51^ The lack of E2 response in male cells in this study could be explained by the single concentration of E2 used, although this concentration was seen to have effects in the study by Feng *et al.*,^52^ or differences in assay conditions.

#### Human cells

In most reported cases, E2 treatment increased proliferation and survival in human ASCs. Roncari and Van found that E2 treatment increased the cell count and proliferation rate of adipocyte precursors but did not alter cell size.^53^ Furthermore, E2 treatment of female ASCs increased cell proliferation and decreased apoptosis of cells cultured in serum-free media.^54^ Hong *et al.* reported that E2 stimulation of female ASCs increased proliferation during osteogenesis but not during adipogenesis.^55^ Anderson *et al.* explored the effects of multiple concentrations of E2 on preadipocytes isolated from subcutaneous and omental tissue of male and female humans. They found that E2 treatment increased proliferation in all tissues. Time to maximal proliferation varied by sex and tissue type, occurring later in men and omental tissue.^56^

While exploring the effects of ASCs isolated from subcutaneous adipose tissue from the femoral and abdominal regions of postmenopausal women treated with E2 and placebo, Cox-York *et al.* found that E2 treatment significantly increased the differentiation potential of femoral stem cells but not abdominal stem cells.^57^ Ng *et al.* found that there was no difference in the chondrogenic potential of ASCs collected from female humans that were pregnant, premenopause, or menopausal.^58^

Most studies report that E2 treatment improves the differentiation potential of ASCs from humans. E2 treatment of ASCs isolated from females has been found to increase osteogenic^47,55^ and adipogenic^47,54,55^ potential. Sadeghi *et al.*, although, found that E2 treatment of human ASCs (sex not specified) inhibited chondrogenesis.^59^

### In vivo

#### Mouse

Lapid *et al.* found that adipose progenitor cell number and proliferation were increased in ovx mice compared to sham controls. In this study, the authors also generated adipose-lineage ERα-mutant male and female mice and found that they displayed significant reductions in adipose progenitor cell number compared to control mice. The adipogenic potential of adipose progenitor cells was found to be reduced in mutant animals compared to controls, while the ability to differentiate into smooth muscle was increased. These results indicate that ERα is important for adipogenesis and for maintaining an adipose progenitor cell population.^60^ ERβ-mutant animals were not investigated.

#### Human

Studies comparing ASCs collected from females of different reproductive status have shown that cells from pregnant donors proliferate more rapidly than cells from premenopausal or menopausal women,^58^ but that there is not a significant difference in rates between premenopausal and menopausal women.^56,58^ Conversely, Cox-York *et al.* compared ASCs isolated from subcutaneous adipose tissue from postmenopausal women treated with E2 and placebo and found that E2 treatment did not alter proliferation, susceptibility to apoptosis, or expression of ERα or ERβ mRNA.^57^

Studies of the effects of estrogens on ASCs are summarized in Table 2. In most cases, E2-treated ASCs showed increased differentiation potential and proliferation and decreased apoptosis; these are similar trends compared to BM-MSCs.

**Table 2.** Role of Estrogens on Adipose Derived Stem Cell Stemness

Cell type	ASCs					
	Sex	Animal model, age	In vitro, in vivo	Hormone treatment	Response	Study
Mouse cells						
ASCs	M	C57BL/6 mice, 8-week old	*In vitro*	0, 50, 100, 200 nM PPT (ERα agonist), DPN (ERβ agonist), or 182,780 (ER antagonist)	Both agonists increased stem cell proliferation. ERα agonist encouraged wound healing and cell migration. ERα agonist stimulates brown adipogenesis, while ERβ agonist inhibits it.	Zhang *et al.*^49^
Rat cells						
ASCs	F and M	Sprague-Dawley rats, 1-month old	*In vitro*	10^−7^–10^−11^ E2	E2 treatment: increased cell proliferation and myogenic differentiation; treated cells formed a more solid cell layer on electrospun mesh than control cells	Feng *et al.*^50^
Preadipocytes from femoral, epididymal, and parametrial tissue	F and M	Sprague-Dawley rats, age N/A	*In vitro*	10, 100, 1000 nM E2; ovx	E2 treatment: increased preadipocyte growth rate from both ovx and intact females, but not from males; increased GPDH activity in cells from females, but not in males	Dieudonne *et al.*^51^
Human cells						
ASCs	F and M	Human, 35–54-year old	*In vitro*	None	M showed greater osteogenesis compared to F	Aksu *et al.*^25^
ASCs	F and M	Human, 18–71-year old	*In vitro*	None	Many chromosomal segments and individual genes were found to be differentially expressed between the sexes, including some related to immunomodulation, differentiation, and cell-cell or cell-ECM adhesion.	Bianconi *et al*.^26^
Omental adipose-derived precursor cells	F and M	Human, 20–60-year old	*In vitro*	0.5–500 ng/mL E2	E2 treatment: increased adipose-derived precursor cell count and replication; did not alter cell size	Roncari and Van^53^
Preadipocytes from subcutaneous and omental tissue	F and M	Human, pre-and postmenopausal for F	*In vitro*	10^−7^, 10^−8^, 10^−9^ M E2	E2 treatment: increased proliferation in preadipocytes from all sources; time to maximal proliferation varied by sex and tissue type, occurring later in men and omental tissue	Anderson *et al.*^56^
ASCs	F	Human, 45-year old	*In vitro*	10^−8^–10^−11^ M E2	E2 treatment + adipogenic stimulation: enhanced adipogenic potential; did not alter proliferation during adipogenesis E2 treatment + osteogenic stimulation: enhanced osteogenic potential: increased proliferation	Hong *et al.*^55^
ASCs	F	Human, pregnant, premenopause, menopause	*In vitro*	10^−8^ M E2	Hormone status: no difference in chondrogenic potential between groups E2 treatment: no effect on proliferation or ER expression	Ng *et al.*^58^
ASCs	F	Human, 22–30-year old	*In vitro*	10^−6^–10^−10^ M E2	E2 treatment: increased cell proliferation, VEGF production, and adipogenic potential; decreased apoptosis in serum-free media	Luo *et al.*^54^
ASCs	F	Human, age N/A	*In vitro*	100 pM-1 mM E2	E2 treatment: increased osteogenic and adipogenic potential	Strong *et al.*^47^
ASCs	N/A	Human, 25–55-year old	*In vitro*	10^−8^ M E2	E2 treatment: decreased chondrogenesis	Sadeghi *et al.*59
ASCs	F	Human, 45–60-year old (postmenopausal)	*In vivo*	3 × 0.005 mg/14 days E2 patches	E2 treatment: increased differentiation in stem cells obtained from the femoral region but not from the abdominal region	Cox-York *et al.*^57^
Mouse						
White adipose progenitor cells	F and M	C57 mice, age N/A	*In vivo*	None	ERα promotes adipogenic lineage commitment, ERα-mutant mice experience characteristic metabolic symptoms consistent with brown phenotype	Lapid *et al*^60^
Human						
ASCs	F	Human, pregnant, premenopause, menopause	*In vivo*	None	Hormone status: cells from pregnant donors showed a higher proliferation rate than the other groups	Ng *et al.*^58^
ASCs	F	Human, 45–60-year old (postmenopausal)	*In vivo*	3 × 0.005 mg/14 days E2 patches	E2 treatment: did not alter proliferation, susceptibility to TNF-α, or mRNA expression of ERα or β.	Cox-York *et al.*^57^

ASC, adipose-derived stem cell; DPN, diarylpropionitrile; ECM, extracellular matrix; GPDH, glycerol-3-phosphate dehydrogenase; PPT, propyl pyrazole triol.

## Estrogens and MDSCs

### In vitro

#### Mouse cells

Intact hormone status and the presence of functioning ERβ are important for mouse satellite cell function. Satellite cells of ovx mice were found to be impaired in self-renewal and differentiation abilities compared to those collected from control mice.^61,62^ In addition, work with satellite cells isolated from satellite cell-specific conditional ERβ knockout (ERβKO) mice revealed that both male and female ERβKO cells failed to proliferate compared to wild type (WT) control, indicating the importance of ERβ signaling for proliferation of both sexes.

In addition, when single myofibers were isolated from mice and cultured in floating conditions, a common method for studying the activation and function of the associated satellite cells,^63^ myofibers isolated from KO animals generated less satellite cells than WT, but the proportion of proliferative, self-renewing, and differentiation-committed satellite cells was the same. This indicates that ERβ regulates satellite cell proliferation rate but not fate decision.^64^ This study did not investigate the effects of ERαKO; therefore, comparisons between the roles of ERs in these processes cannot be made.

E2 treatment impairs myogenic differentiation in cells isolated from mice. E2 treatment was found to impair mouse myoblast differentiation in both the C2C12 immortalized cell line (sex not specified)^65,66^ and myoblasts isolated from the hind limb muscle of C57BL/6 mice (sex not specified).^65^ In addition, Ogawa *et al.* found that E2 inhibited myogenesis in satellite cells isolated from the hind-limb muscles of neonatal and young female mice.^66^

#### Cow cells

Kamanga-Sollo *et al.* reported that treatment of proliferating satellite cells isolated from steers with 10 nM E2 increased proliferation rate, but only when cells were cultured in 1% insulin-like growth factor (IGF) binding protein 3-free swine serum. No E2-related effect on proliferation was seen in cells cultured in standard swine serum or fetal bovine serum.^67^ This increase in proliferation rate was tied to signaling through ER, insulin-like growth factor receptor, MEK1, and PI3K/protein kinase B (AKT) pathways.^68^

In another study, Kamanga-Sollo *et al.* reported that the effects of E2 on IGF-1 mRNA levels and proliferation are mediated through different mechanisms, with IGF-1 mRNA levels, which they had previously shown to increase with E2 treatment,^67^ likely being controlled through G protein-coupled receptor 30 (GPR30).^69^ Further studies of fused bovine satellite cells isolated from steers revealed that treatment with E2 caused a concentration-dependent increase in protein synthesis and decrease in protein degradation. Both of these effects were linked to ERα and/or ERβ.^70^

#### Human cells

Li *et al.* found that E2 treatment stimulated proliferation in human male smooth muscle progenitor cells derived from both embryonic stem cell lines and induced pluripotent stem cells but not in female cells of either population. In addition, E2 treatment increased myogenic gene markers and suppressed extracellular matrix (ECM) degradation in female cells but not in male cells from both sources.^24^

### In vivo

#### Mouse

Collins *et al.* found that ovx mice had fewer satellite cells in multiple types of muscle samples compared to control mice and that E2 treatment rescued satellite cell numbers.^61^ Conversely*,* Kitajima and Ono did not observe a decrease in the number of satellite cells in the extensor digitorum longus of ovx mice compared to control mice, but they did find an increase in the number of myonuclei per fiber.^62^

In agreement with *in vitro studies*, E2 treatment has been found to impair myogenesis *in vivo*. Ogawa *et al.* report that E2 treatment of ovx mice decreased the ratio of skeletal muscle mass to body weight and increased ubiquitin-specific peptidase 19 expression, indicating that E2 inhibits myogenesis *in vivo*.^66^ A study of satellite cell-specific conditional ERβKO mice by Seko *et al.* revealed that ERβ is important for regulation of postnatal muscle growth but not muscle maintenance in females but not males.^64^ ERαKO animals were not investigated in this study.

#### Rat

E2 treatment affects the number of satellite cells found in rat muscles after exercise. Tiidus *et al.* found that the total number of satellite cells in the soleus and white vastus of Sprague-Dawley rats increased after exercise and that this effect was enhanced by E2 treatment. E2 treatment had no effect on the number of satellite cells in nonexercised rats.^71^ Further work showed that this increase was seen in numbers of total, activated, and proliferating satellite cells.^72^ It was found that ERα likely plays a primary role in this increase.^73,74^ Mangan *et al.* also found that the PI3K/AKT pathway was implicated in a similar increase in satellite cell numbers in the soleus and white gastrocnemius muscles of Sprague-Dawley rats.^75^

E2 treatment can also stimulate muscle regeneration after injury. Greater satellite cell activation and proliferation plus muscle regeneration compared to ovx control was seen in ovx Wistar rats treated with E2 and the ERβ-selective agonist 8β-VE2 but not with the ERα agonist 16α-LE2, indicating a role for ERβ in muscle regeneration.^76^

#### Human

Collins *et al.* studied needle muscle biopsies taken from the vastus lateralis of the same women when they were perimenopausal and again after they were postmenopausal and found that the number of satellite cells collected from each patient decreased with change in menopausal state.^61^

Overall, MDSCs from both males and females are affected by E2 treatments. Cells treated with E2 saw an increase in proliferation and cell numbers. Osteogenic differentiation potential was increased with E2 treatment, while myogenic differentiation potential was decreased. Studies of MDSCs are summarized in Table 3.

**Table 3.** Role of Estrogens on Muscle Derived Stem Cell Stemness

Cell type	MDSCs					
	Sex	Animal model, age	In vitro, in vivo	Hormone treatment	Response	Study
Mouse cells						
MDSCs	F and M	C57BL/6J mice, 3-week old	*In vitro*	None	M: more rapid and greater extent of osteogenesis	Corsi *et al.*^27^
MDSCs	F and M	C57.BL10 mice, 3-week old	*In vitro*	None	M: undergo chondrogenesis more effectively and produce larger pellets with richer ECM; chondrogenic potential maintained in long term culture	Matsumoto *et al.*^14^
ERβKO satellite cells from the extensor digitorum longus	F and M	Mice with ERβKO satellite cells, 6- and 20-week old	*In vitro*	None	M and F ERβKO satellite cells: failed to proliferate compared to WT cells; proportion of proliferative, self-renewing, and differentiation-committed cells not effected	Seko *et al.*^64^
C2C12 cell line (immortalized mouse myoblasts)	N/A	Mouse, age N/A	*In vitro*	0, 0.1, 1, 10, 100, 1000 nM E2; 10 nM PPT; 10 nM DPN; 1 μM ICI 182,780	E2 treatment: inhibited myogenesis; increased USP19 mRNA PPT (ERα agonist) treatment: inhibited myogenesis DPN (ERβ agonist) treatment: no change in myogenesis E2 and ICI 182,780 (ER antagonist) cotreatment: E2 inhibitory effects blocked	Ogawa *et al.*^66^
Satellite cells from hind limb muscles	F	Kwl:ddY mice, 3–5-day old (neonatal) or 7–8-week old (young)	*In vitro*	0, 0.1, 1, 10, 100, 1000 nM E2	E2 treatment: inhibited myogenesis; increased USP19 mRNA and protein levels in a dose-dependent manner	Ogawa *et al.*^66^
C2C12 cell line (immortalized mouse myoblasts)	N/A	Mouse, age N/A	*In vitro*	0, 0.01, 0.1, 0.5, 1 μM E2	E2 treatment: impaired myoblast differentiation	Go *et al.*^65^
Myoblasts isolated from the hind limb muscle	N/A	C57BL/6 mice, 1-month old	*In vitro*	1 μM E2	E2 treatment: impaired myoblast differentiation	Go *et al.*^65^
Cow cells						
Proliferating satellite cells from semimembranous muscle	M	Castrated cattle (Steer)	*In vitro*	0.001, 0.01, 0.1, 1, 10 nM E2	Treatment with: 0.001 nM E2: increase in ERα and IGFBP-3 mRNA 0.01–10 nM E2: increase in IGF-1 mRNA 10 nM E2: increase in proliferation rate	Kamanga-Sollo *et al.*^67^
Proliferating satellite cells from semimembranous muscle	M	Castrated cattle (Steer)	*In vitro*	10 nM E2; 10 nM ICI 182,780; 10 μg/mL JB1; 0, 20, 100, 500 μM PD98059; 0, 100, 500, 1000 nM wortmannin	E2 treatment: increased IGF-1 mRNA in the presence of FBS not SS; increased proliferation rate in the presence of SS not FBS; proliferation increase blocked by ICI 182,780 (ER antagonist), JB1 (competitive inhibitor of IGFR-1), PD980059 (MEK1 inhibitor), and wortmannin (PI3K/Akt pathway inhibitor)	Kamanga-Sollo *et al.*^68^
Proliferating satellite cells from semimembranous muscle	M	Castrated cattle (Steer)	*In vitro*	10 nM E2; 10, 100 nM ICI 182,780; 10, 100 nM G1; 100, 1000 nM BSA-E2	ICI 182,780 (ER antagonist) treatment: increase in IGF-1 mRNA G1 (GPR30 agonist) or BSA-E2 (cell impermeable E2) treatment: increase in IGF-1 mRNA, no change in proliferation rate	Kamanga-Sollo *et al.*^69^
Fused satellite cells from semimembranous muscle	M	Castrated cattle (Steer)	*In vitro*	0.1, 1, 10 nM E2, 100 nM ICI 182,780, 100 nM G1 (GPR30 agonist)	E2 treatment: concentration-dependent increase in protein synthesis; decrease in protein degradation; blocked by ICI 182,780 cotreatment G1 treatment: no change in protein synthesis or degradation	Kamanga-Sollo *et al.*^70^
Human cells						
MDSCs	F and M	Human, 12–92-year old	*In vitro*	None	M: undergo chondrogenesis and osteogenesis more than F	Scibetta *et al.*^16^
Smooth muscle progenitor cells from embryonic stem cell line	F and M	Human, blastocyst stage embryo	*In vitro*	0, 0.1, 1.0, 10 nM E2	E2 treatment: F: increased myogenesis and reduced ECM degradation M: increased proliferation	Li *et al.*^24^
Smooth muscle progenitor cells from induced pluripotent stem cells	F and M	Human, 28–45-year old	*In vitro*	0, 0.1, 1.0, 10 nM E2	F express more ERβ; F and M express equivalent ERα E2 treatment: F: increased myogenesis and decreased ECM degradation M: increased proliferation	Li *et al.*^24^
Mouse						
MDSCs	F and M cells and hosts	C57BL/6J mice, age N/A	*In vivo*	None	M hosts: greater bone formation area and density regardless of sex of implanted cells	Corsi *et al.*^27^
MDSCs	F and M	C57 mice, 3-week old	*In vivo*	None	F: regenerated skeletal muscle more efficiently M: faster MDSC pool depletion	Deasy *et al.*^28^
MDSCs	F and M cells, F hosts	C57.BL10 mice, 3-week old (cells); nude rats, 12-week old (hosts)	*In vivo*	None	M cells: greater cartilage regeneration in osteochondral defect	Matsumoto el al.^14^
MDSCs isolated from lower limbs	M cells; M and F hosts	C57BL/6J mice (cells); C57BL/6J mice, 12-week old (hosts)	*In vivo*	ovx/castrated	Ectopic bone formation: M hosts (unaltered and castrated) formed more bone than both F (unaltered and ovx) Cranial defect healing: M hosts formed more bone than F	Meszaros *et al.*^15^
ERβKO satellite cells	F and M	Mice with ERβKO satellite cells, 6- and 20-week old	*In vivo*	None	F ERβKO mice: reduction in muscle weight and regeneration after injury compared to control; not exacerbated by ovx M ERβKO mice: no change from control	Seko *et al.*64
Satellite cells from gastrocnemius and soleus muscles	F	Kwl:ddY mice, 7-week old	*In vivo*	0.1 mg/kg estradiol valerate; ovx	E2 treatment of ovx animals: decreased ratio of skeletal muscle mass to body weight; increased USP19 expression	Ogawa *et al.*^66^
Satellite cells from extensor digitorum longus	F	C57BL/6 mice, 6-week old	*In vivo*, *in vitro*	0.01 mg/60 days slow-release E2 pellet; ovx	ovx: change in number of myonuclei per fiber, not number of satellite cells per fiber; muscles did not regenerate well after injury; satellite cells deficient in self-renewal and differentiation	Kitajima and Ono^62^
Satellite cells from diverse muscles	F	C57/BL6 and Pax7-ZsGreen mice, 3–4 month old	*In vivo*	0.18 mg/60 days slow release E2 pellet; ovx	ovx: fewer satellite cells; satellite cells impaired in self-renewal and differentiation, higher apoptosis E2 treatment: restored satellite cell number in ovx	Collins *et al.*^61^
Rat						
Satellite cells from the soleus and white vastus	M	Sprague-Dawley, 11-week old	*In vivo*	25 mg/21 days E2 pellet	E2 treatment with exercise: increase in satellite cell number compared to exercise alone	Tiidus *et al.*^71^
Satellite cells from the soleus and white vastus	F	Sprague-Dawley rats, 11-week old	*In vivo*	0.25 mg/21 days E2 pellet; ovx	ovx animals with E2 treatment and exercise: increase in total, activated, and proliferating satellite cells compared to exercise alone	Enns and Tiidus^72^
Satellite cells from the soleus and white vastus	F	Sprague-Dawley rats, 11-week old	*In vivo*	0.25 mg/21 days E2 pellet; 5 mg/kg ICI 182,780 (ER antagonist); ovx	ovx animals with E2 treatment and exercise: increase in total, activated, and proliferating satellite cells compared to exercise alone; results blocked by ER antagonist	Enns *et al.*^73^
Satellite cells from the soleus and white vastus	F	Sprague-Dawley rats, 11-week old	*In vivo*	0.25 mg/21 days E2 pellet; 0.5 mg/day PPT (ERα agonist); ovx	ovx animals with exercise and E2 or PPT treatment: increase in total, activated, and proliferating satellite cells compared to exercise alone	Thomas *et al.*^74^
Satellite cells from gastrocnemius	F	Wistar rats, 8-week old	*In vivo*	40 μg/kg bw/d E2; 10 μg/kg bw/d 16α-LE2; 100 μg/kg bw/d 8β-VE2; ovx	E2 and 8β-VE2 (ERβ agonist) treatment in ovx animals: greater satellite cell activation and proliferation and muscle regeneration seen after injury compared to ovx control	Velders *et al.*^76^
Satellite cells from the soleus and white gastrocnemius	F	Sprague-Dawley rats, 8-week old	*In vivo*	0.25 mg/21 days slow release E2 pellet; ovx	ovx animals with E2 treatment and exercise: increase in total, activated, and proliferating satellite cells compared to exercise alone; results linked to PI3K/Akt pathway	Mangan *et al.*^75^
Human						
Satellite cells from the vastus lateralis	F	Human, peri- to postmenopause	*In vivo*	None	Samples were taken from the same women at peri- and postmenopause. Satellite cell number decreased.	Collins *et al.*^61^
MDSCs	F and M cells, male hosts	Human, 12–92-year old (cells), ICR-SCID mice, 8-week old (host)	*In vivo*	None	M cells: better able to regenerate bone	Scibetta *et al.*^16^

AKT, protein kinase B; ERβKO, ERβ knockout; FBS, fetal bovine serum; GPR30, G protein-coupled receptor 30; ICI, Imperial Chemical Industries; IGF, insulin-like growth factor; MDSC, muscle-derived stem cell, SS, swine serum; WT, wild type.

## Estrogens and Periodontal Ligament Stem Cells

### In vitro

#### Rat cells

Periodontal ligament stem cells (PDLSCs) isolated from ovx rats have decreased osteogenic potential compared to sham controls, and E2 treatment is able to improve this.^52,77^ Zhang *et al.* linked both ERα and ERβ to the E2-dependent increase in osteogenic potential of PDLSCs from rats.^77^

#### Human cells

E2 treatment is generally beneficial for human PDLSCs. Ou *et al.* found that treatment with 0.1 μM E2 increased proliferation rates compared to control and improved the proliferation, stemness, and both osteogenic and adipogenic potential of PDLSCs in long-term culture. Treatment with E2 also increased the proportion of cells in the G2/M + S phase of the cell cycle and increased expression of stemness-related genes through the PI3K/AKT pathway.^78^ Similarly, Pan *et al.* found that E2 treatment of PDLSCs isolated from female humans increased osteogenic potential of PDLSCs in a dose-dependent manner. This effect was linked to both ERα and ERβ.^79^ In addition, E2 treatment increased osteogenesis in PDLSCs from adolescents through activation of the Wnt/β-catenin pathway.^80^

### In vivo

The hormone status of rats affects the properties of their PDLSCs. Zhang *et al.* reported that periodontal ligaments from ovx Sprague-Dawley rats contained more PDLSCs than those from control animals. Furthermore, cells collected from the different groups showed different metabolic kinetics, with ovx cells being more metabolically active at early time points but the two groups showing equivalent levels by day 11.^77^ Similarly, E *et al.* found that PDLSCs isolated from ovx rats and grown in 2D culture had higher proliferation rates than cells from sham animals or ovx cells treated with E2. They additionally studied PDLSCs isolated from ovx and sham rats and grown on 3D nano-hydroxyapatite/collagen/poly(L-lactide) scaffolds with and without E2 treatment. When the seeded scaffolds were implanted in SCID mice, all cell types led to new bone growth after 12 weeks, with cells from ovx rats generating the least.^52^

The effect of E2 treatment on the proliferation of PDLSCs is dependent on the environment, as proliferation increased *in vitro*, but decreased *in vivo*. E2 treatment's effects on differentiation capabilities are similar to what has been seen in cell types discussed previously. Osteogenic and adipogenic differentiation improved with the treatments. Studies of connective tissue-derived stem cells, including PDLSCs, are summarized in Table 4.

**Table 4.** Role of Estrogens on Connective Tissue Derived Stem Cell Stemness

Cell type	PDLSCs					
	Sex	Animal model, age	In vitro, in vivo	Hormone treatment	Response	Study
Rat cells						
PDLSCs	F	Sprague-Dawley rats, 3-month old	*In vitro*	10^−7^ M E2	E2 treatment: increased osteogenic potential through both ERα and ERβ.	Zhang *et al.*^77^
PDLSCs	F	Sprague-Dawley rats, 3-month old	*In vitro*	10^−7^ M E2; ovx	Cells from ovx rats: higher proliferation rates and lower osteogenic potential than cells from sham or ovx cells treated with E2; cells from all groups grew well on nHAC/PLA scaffold, although cells from ovx rats had lower osteogenic potential.	Ling-Ling *et al.*^52^
Human cells						
PDLSCs	F	Human, 18, 19, and 22-year old	*In vitro*	10^−7^, 10^−8^, 10^−9^ M E2	E2 treatment: increased osteogenic potential in a dose-dependent manner; both ERα and ERβ were important for osteogenic differentiation.	Pan *et al.*^79^
PDLSCs	F and M	Human, 18–20-year old	*In vitro*	10^−6^, 10^−7^, 10^−8^ M E2	Treatment with 10^−7^ M E2: increased proliferation rates, proportion of cells in G2/M+S phase of the cell cycle, and expression of stemness-related genes; the PI3K/AKT pathway was involved E2 treatment in general: improved the proliferation, stemness, and differentiation potential of cells in long-term culture.	Ou *et al.*78
PDLSCs	N/A	Human, 12–16-year old	*In vitro*	10^−7^ M E2	E2 treatment: increased osteogenesis through activation of the Wnt/β-catenin pathway	Jiang *et al.*^80^
Rats						
PDLSCs	F	Sprague-Dawley rats, 3-month old	*In vivo*	ovx	ovx animals: contain more PDLSCs; proliferate faster but decrease sooner	Zhang *et al.*^77^
PDLSCs	F	Sprague-Dawley rats, 3-month old	*In vivo*	10^−7^ M E2; ovx	In seeded nHAC/PLA scaffolds implanted into SCID mice, all cell types led to new bone growth, with cells from ovx rats generating the least	Ling-Ling *et al.*^52^
TDSCs						
TDSCs	M	C57BL/6J mice, 6-month old	*In vivo*	None, but ERβ^−/−^ mice compared to WT	Achilles tendon injury model: WT: more ERβ+ cells found in injured than noninjured animals ERβ^−/−^: lower cell density and higher adipocyte and blood vessel accumulation in scar site than WT	Bian *et al.*^81^
TDSCs	M	Sprague-Dawley rats, 6-week old	*In vitro*	10^−5^, 10^−7^, 10^−9^ M LY3201 (ERβ agonist)	Treatment with 10^−7^ M LY3201: promoted cell proliferation; inhibited adipogenesis; other concentrations had no effect.	Bian *et al.*^81^
CPCs						
CPCs	F and M	Human, with OA	*In vitro*	0.02 or 0.15 ng/mL E2	F: greater percentage of cells expressed ERz and ERβ E2 treatment: F and M: increased ERα and decreased ERβ expression F: increased chondrogenesis	Koelling and Miosge^82^
FCSC						
FCSCs	M	New Zealand White rabbits, 12-week old	*In vivo*	0.1 mL of 100 ng/mL Sost once weekly for 7 weeks	Sclerostin (Wnt pathway inhibitor) treatment after post-traumatic OA induction: increased FCSC number in TMJ superficial zone; decreased joint damage; reduced joint swelling	Embree *et al.*^83^
FCSCs	F	C57BL/6 mice, 3- or 13-week old	*In vivo*	0.01 mg/60 days E2 pellet; ovx	E2 treatment in: Young mice: promoted chondrogenesis through ERα through upregulation of Sost Mature mice: promoted chondrogenesis and anabolic gene expression through ERα	Robinson *et al.*^84^

CPC, chondrogenic progenitor cell; FCSC, fibrocartilage stem cell; nHAC/PLA, nano-hydroxyapatite/collagen/poly(L-lactide); OA, osteoarthritis; PDLSCs, periodontal ligament stem cells; SCID, severe combined immunodeficient; Sost, sclerostin; TDSC, tendon-derived stem cell; TMJ, temporomandibular joint.

## Estrogens and Tendon-Derived Stem Cells

Bian *et al.* studied tendon-derived stem cells (TDSCs) isolated from male Sprague-Dawley rats. Treatment of TDSCs with 0.1 μM of the ERβ agonist LY2301 promoted cell proliferation and inhibited adipogenesis, but no effect was seen at other concentrations. Bian *et al.* also explored the importance of ERβ on Achilles tendon healing in mice using an ERβ^−/−^ male mouse wound model. The authors reported that more ERβ+ cells were present in the scar of injured WT mice 8 days after injury than in noninjured control mice. ERβ^−/−^ mice had lower cell density and higher adipocyte and blood vessel accumulation in the scar site compared to WT. The low cell density was the result of higher levels of apoptosis and lower levels of cell proliferation. These results indicate that the absence of ERβ results in inferior wound healing.^81^ ERα^−/−^ mice were not included in this study, so direct comparisons between the roles of these two receptors cannot be made.

## Estrogens and Chondrogenic Progenitor Cells

Koelling and Miosge found that E2 treatment of chondrogenic progenitor cells isolated from the cartilage of male and female humans with osteoarthritis (OA) had differing effects depending on subject sex and E2 concentration. An increase in chondrogenesis was seen in females after treatment with 0.07 nM E2, but the effects were less clear in males and in both sexes at 0.55 nM E2. ERα and ERβ were both expressed in a greater percentage of female cells than male cells, and E2 treatment caused an increase in the expression of ERα in both sexes. Treatment with 0.07 nM E2 caused no effect in female cells and an increase in ERβ expression in males, while 0.55 nM E2 caused ERβ expression to decrease in both sexes.^82^

## Estrogens and Fibrocartilage Stem Cells

Embree *et al.* have characterized fibrocartilage stem cells (FCSCs), located in the superficial zone of the temporomandibular joint (TMJ) articular fibrocartilage, that have been shown to have differentiation capabilities similar to other mesenchymal stromal cell sources.^83^ This population of cells requires inhibition of the canonical Wnt signaling pathway, such as through sclerostin (Sost), to maintain the stem cell pool and promote tissue repair after injury. E2 signaling through ERα has been shown to promote early evidence of new tissue formation in an ovx model with the effects being mediated by upregulation of Sost.^84^ As such, it is possible that E2 promotes FCSC self-renewal and promotes tissue repair after injury in the TMJ by inhibiting canonical Wnt signaling.

## Summary of Findings and Potential Mechanisms

The hope for the use of stem cells as therapies for many diseases and illnesses has driven the scientific community to study their safety and efficacy. Understanding their innate properties—proliferative abilities, differentiation potential, etc.—is vital to their potential future use in the realm of tissue engineering and regenerative medicine. Also key to our understanding: the role that sex plays in cell behavior and response. As shown in this review, there are inherent differences between cells from male and female donors across *multiple species*. Male cells were found to have stronger osteogenic, chondrogenic, and adipogenic differentiation capabilities than their female counterparts. Although proliferation was typically not found to be different between male and female cells, more cells were found in male hosts than in females.

With some sex-based differences tied to estrogens, treatment of stem cells with estrogens like E2 has been investigated by many, with the hope of improving stem cell capabilities. A generalized summary of their findings can be seen in Figure 3. Across *multiple species* and cell types, cells treated with E2 were found to increase proliferation and decrease apoptosis. Differentiation of the stem cells was also affected, with osteogenic potential often increasing and adipogenic, chondrogenic, and myogenic potentials varying depending on the cell type. With a better understanding of the differences between male and female cells, and the differences in their responses to E2 treatment, stem cells can be better utilized in the fields of tissue engineering and regenerative medicine.

**FIG. 4.**
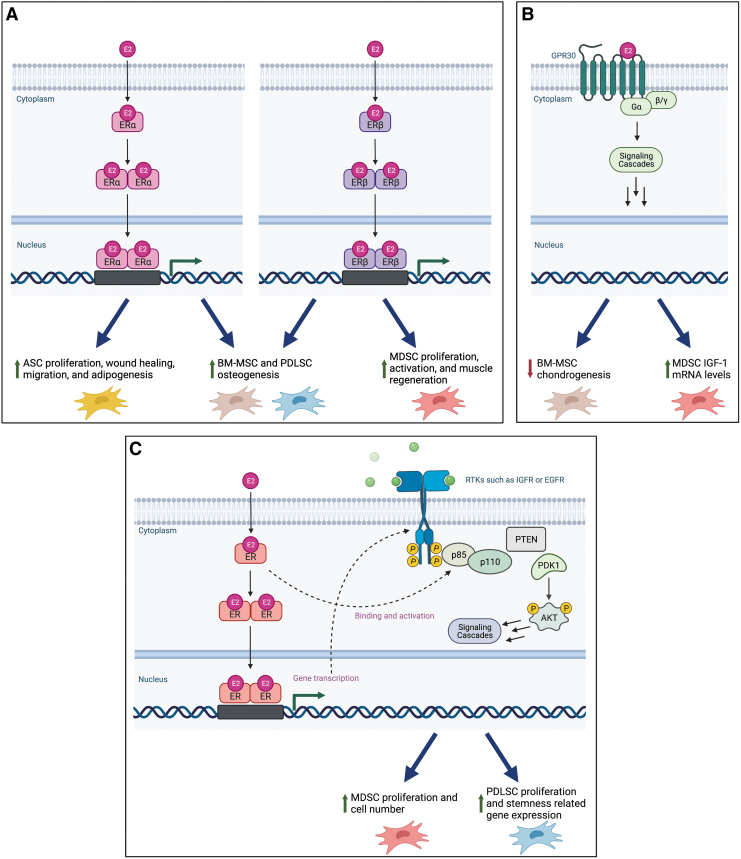
Summary of pathways linked to effects of E2 on stem cells of the musculoskeletal system. **(A)** Many effects have been linked to canonical estrogen receptors α and/or β. The canonical estrogen signaling pathway is pictured featuring homodimerization, although not all the listed effects have been linked exclusively to this pathway and the types of dimers formed have not been investigated. Effects seen in studies that did not investigate both receptors were omitted. **(B)** Other cell responses have been linked to membrane-bound estrogen receptors such as GPR30 rather than canonical estrogen receptors. **(C)** The PI3K/AKT pathway has been linked to several cell responses to estrogen treatment. This pathway can be activated by estrogen signaling through routes, including estrogen-stimulated promotion of transcription of PI3K/AKT pathway components and the direct binding of the estrogen/estrogen receptor complex to the p85 subunit of PI3K/AKT, as previously reviewed.^92^ Figure created using BioRender.com AKT, protein kinase B; EGFR, epidermal growth factor receptor; ER, estrogen receptor; GPR30, G protein-coupled receptor 30; IGF-1, insulin-like growth factor 1; IGFR-1, insulin-like growth factor receptor-1; PDK1, phosphoinositide-dependent kinase-1; PI3K, phosphoinositide 3-kinase; PTEN, phosphatase and tensin homolog; RTK, receptor tyrosine kinase. Color images are available online.

Many of these studies also worked to identify the receptors and pathways involved in the observed responses to E2 treatment. Some common themes emerged, which are summarized in Figure 4 and below with further context. Much of this effort focused on identifying which estrogen receptor is responsible for the various changes caused by E2 treatment. Estrogens modulate transcription through both classical and nonclassical pathways. In the classical pathway, an estrogen binds to ERα or ERβ resulting in a receptor conformational change, receptor dimerization, and translocation into the nucleus.^85^ The receptor complex then typically binds to DNA sequences termed estrogen response elements and acts as an enhancer, recruiting coregulators to promote gene transcription.^86,87^ In the nonclassical pathway, estrogens bind to membrane-associated ERs such as GPR30 or the classical ERs interact with other transcriptional pathways through protein–protein interactions.^88,89^

ERα and ERβ have similar structures and are both composed of multiple domains (A–F). The amino acid sequence homology between the two receptors varies across the domains and is highest for the DNA binding domain (C domain) and the ligand binding domain (E domain). The two receptors have overlapping yet distinct and often opposite downstream effects. These differences are not fully understood, but are due, in part, to differences in their F domains^90^ and preferential use of different coregulators.^91^

Both ERα and ERβ have been implicated in increases in osteogenic potential of BM-MSCs^33^ and PDLSCs,^77,79^ so it would appear that genes involved in this process are regulated by both receptors. Other responses were found to be ER specific or were only investigated in terms of a single receptor.

In the reviewed articles, ERα was frequently seen to play important roles in ASCs, while ERβ signaling was studied in the context of muscle satellite cells. ERα has been linked to ASC adipogenesis and population maintenance,^60^ as well as E2-stimulated increases in satellite cell number, although a similar role for ERβ was not fully excluded in these studies.^73,74^ Furthermore, the ERα agonist PPT increased ASC proliferation, wound healing, migration, and brown adipogenesis, while the ERβ agonist DPN had lesser effects on proliferation, no effect on wound healing or migration, and inhibited brown adipogenesis.^49^

ERβ serves important roles in muscle satellite cells. Treatment of injured ovx Wistar rats with the ERβ-selective agonist 8β-VE2 but not with the ERα agonist 16α-LE2 led to greater satellite cell activation and proliferation plus muscle regeneration compared to ovx control.^76^ In addition, satellite cells isolated from satellite cell-specific ERβKO mice revealed that both male and female ERβKO cells failed to proliferate compared to WT control. In addition, isolated myofibers from KO animals cultured in floating conditions generated less satellite cells than WT, but the proportion of proliferative, self-renewing, and differentiation-committed cells was the same. This indicates that ERβ regulates satellite cell proliferation but not fate decision.^64^ Furthermore, Seko *et al.* used KO studies to show that ERβ is important for regulation of postnatal muscle growth but not muscle maintenance in female but not male mice.^64^ ERαKO animals were not investigated in these two studies, limiting the conclusions that can be drawn.

Some of the effects of E2 treatment have also been linked to membrane-associated ERs. For example, the inhibition of chondrogenesis in male human BM-MSCs in 3D culture by E2 was tied to membrane-associated ERs such as GPR30 by Jenei-Lanzal *et al.*^44^ In addition, Kamanga-Sollo *et al.* found that although E2-stimulated increases in bovine satellite cell proliferation were mediated through classical ERs, increases in IGF-1 mRNA levels were controlled by GPR30.^69^

The PI3K/AKT pathway has been tied to E2 treatment-induced increases in proliferation rate^68^ and cell number^75^ of satellite cells and proliferation rate and expression of stemness-related genes in PDLSCs.^78^ These results are not surprising given that PI3K/AKT activation leads to cell proliferation and that this pathway is closely linked to and can be activated by ER signaling.^92^ Furthermore, this pathway has been linked to the maintenance of the undifferentiated state of human embryonic stem cells and to differentiation of many types of stem cells, including ADSCs and PDLSCs, as discussed in a review by Ramazzotti *et al.*^93^

Other pathways have been linked to the effects of estrogen treatment as well. E2 treatment increased osteogenesis in PDLSCs from adolescents through activation of the Wnt/β-catenin pathway.^80^ Another study revealed that E2 treatment decreased chondrogenic potential in female rat BM-MSCs and activated the MAPK pathway.^37^ In addition, Wu *et al.* found that E2 control of proliferation and senescence of BM-MSCs were linked to the JAK2/STAT3 pathway.^30^

Although the studies summarized above have contributed much to the understanding of estrogen signaling in stem cells, there is still work to be done. Many of the studies into the roles of ERα and ERβ investigated only one of the receptors, not both. Future studies of these mechanisms should focus on both receptors to allow for full elucidation of the differing roles of the canonical ERs. In addition, more data are needed to understand the mechanisms behind these differing roles. Differences in receptor expression levels dependent on tissue source and donor sex, differences in ER homo- versus hetero-dimerization, and differences in downstream gene targets are possible explanations that could be further pursued. In addition, little information is available on the roots of the sex differences seen in stem cells. It is possible that these differences are based on sexual dimorphisms in ER receptor expression levels or preferred signaling modalities, but studies must be performed to test these hypotheses.

## Key Challenges, Critical Issues, and Unanswered Questions

Stem cell therapies were initially expected to revolutionize the treatment of musculoskeletal disorders. This optimism has been tempered over the years by the lack of convincing preclinical and clinical trial data and unsupported claims made by groups targeting uninformed consumers. This led the Food and Drug Administration (FDA) to publish a cautionary article in 2017 calling for a reliance on sound science in the field of stem cell therapy^94^ in addition to multiple statements on the FDA website warning consumers about the existence and risk of unapproved stem cell therapies.

The enthusiasm and promise of stem cell therapies have not been completely cast aside, though. Hematopoietic progenitor cells are FDA approved to treat disorders that affect the production of blood. As of July 2021, there are 124 active studies listed on clinicaltrials.gov investigating stem cells for the treatment of musculoskeletal diseases. Multiple reviews have been published highlighting the state of the art in stem cell therapies, including some focused on musculoskeletal applications.^95–98^ However, as highlighted in this review, the role of stem cell sex must be considered.

There are several critical issues and key unanswered questions that must guide the future studies in this area. While the role of estrogen signaling is complex and not well understood, estrogens are critical as stem cells from ovx animals tend to be less robust and functional. Given the importance of stem cell sex to their properties and the outcomes of regenerative therapy, it is imperative that more studies state the sex of the subjects used and perform sex comparative studies.

Furthermore, the challenges in making comparisons between studies for this review highlight the need for standardization. Media components such as phenol red, an estrogen mimetic compound, growth factors, and endogenous estrogens in fetal bovine serum can mask the effects of E2 treatment and cause inconsistent results between studies. In addition, differences are seen in the effects of estrogens based on the concentrations used, as well as variabilities between tissue sources, donors within a species, and donors of separate species. This greatly complicates the application of knowledge gained from one model onto another. The standardization of conditions and determination of an ideal model for studying the effects of E2 on musculoskeletal tissue are needed to produce results relevant to human disease. Furthermore, there is a need for more physiological tissue models for *in vitro* and *ex vivo* testing to parse out E2 effects in a more controlled manner with the ability to include mechanical effects, which do not affect males and females equally.

The above data highlight the importance of sex and estrogens for many key stem cell properties and emphasize the potential for improving tissue engineering and regenerative therapy. It is possible that more work in this area, beginning with a conscious effort by researchers to state the sex of research subjects and perform more comparative studies, could allow stem cell therapies to revolutionize tissue engineering as originally hoped. Furthermore, properly controlled studies of the effects of E2, other estrogens, and selective estrogen receptor modulators should be carried out to establish a deeper understanding of their potential roles in regenerative therapy.
